# Perceived Stress Is Differentially Related to Hippocampal Subfield Volumes among Older Adults

**DOI:** 10.1371/journal.pone.0154530

**Published:** 2016-05-04

**Authors:** Molly E. Zimmerman, Ali Ezzati, Mindy J. Katz, Michael L. Lipton, Adam M. Brickman, Martin J. Sliwinski, Richard B. Lipton

**Affiliations:** 1 Department of Psychology, Fordham University, Bronx, New York, United States of America; 2 Department of Neurology, Albert Einstein College of Medicine, Bronx, New York, United States of America; 3 Department of Radiology, Albert Einstein College of Medicine, Bronx, New York, United States of America; 4 Department of Neuroscience, Albert Einstein College of Medicine, Bronx, New York, United States of America; 5 Gruss Magnetic Resonance Research Center, Albert Einstein College of Medicine, Bronx, New York, United States of America; 6 Taub Institute for Research on Alzheimer’s Disease and the Aging Brain, Columbia University College of Physicians and Surgeons, New York, New York, United States of America; 7 Department of Human Development and Family Studies, Pennsylvania State University, University Park, Pennsylvania, United States of America; 8 Department of Epidemiology and Population Health, Albert Einstein College of Medicine, Bronx, New York, United States of America; 9 Department of Psychiatry and Behavioral Sciences, Albert Einstein College of Medicine, Bronx, New York, United States of America; Southwest University, CHINA

## Abstract

**Introduction:**

Chronic exposure to stress has been shown to impact a wide range of health-related outcomes in older adults. Despite extensive animal literature revealing deleterious effects of biological markers of stress on the dentate gyrus subfield of the hippocampus, links between hippocampal subfields and psychological stress have not been studied in humans.

This study examined the relationship between perceived stress and hippocampal subfield volumes among racially/ethnically diverse older adults.

**Methods and Materials:**

Between July 2011 and March 2014, 116 nondemented participants were consecutively drawn from the Einstein Aging Study, an ongoing community-based sample of individuals over the age of 70 residing in Bronx, New York. All participants completed the Perceived Stress Scale, Geriatric Depression Scale, and underwent 3.0 T MRI. FreeSurfer was used to derive total hippocampal volume, hippocampal subfield volumes (CA1, CA2/CA3, CA4/Dentate Gyrus (CA4/DG), and subiculum), entorhinal cortex volume, whole brain volume, and total intracranial volume.

**Results:**

Linear regression analyses revealed that higher levels of perceived stress were associated with smaller total hippocampal volume (β = -0.20, t = -2.40, p = 0.02), smaller CA2/CA3 volumes (β = -0.18, t = -2.24, p = 0.03) and smaller CA4/DG volumes (β = -0.19, t = -2.28, p = 0.03) after controlling for total intracranial volume, age, gender, and race. These findings remained unchanged after removal of individuals with clinically significant symptoms of depression.

**Discussion:**

Our findings provide evidence of a relationship between a direct indicator of psychological stress and specific hippocampal subfield volumes in elderly individuals. These results highlight the importance of clinical screening for chronic stress in otherwise healthy older adults.

## Introduction

Many biological and environmental factors are thought to contribute to age-related individual differences in health. Cumulative exposure to stress, characterized as a biological, psychological, or behavioral response to a real or perceived threat, has been shown to impact a wide range of health outcomes in older adults[1–5]. Although the stress response is adaptive or even protective on a short-term basis, prolonged stress may produce deleterious effects on health, including brain changes[6–9]. Stress can be measured at the level of environmental demands (e.g., major life events or daily hassles), at the level of perceived stress, and by examining biological consequences of stress on the hypothalamic pituitary axis (HPA) or the autonomic nervous system (e.g., cortisol). Although some research has linked markers of biological stress to brain structure, fewer studies have examined direct measures of psychological stress and their relationships to the brain. Here, we focus on the hippocampus and the entorhinal cortex because they are particularly sensitive to normal aging effects and age-related pathology. In addition, the hippocampus is part of a connected brain network that regulates the HPA axis that promotes adaptation to stressors (allostasis) and, when compromised, contributes to maladaptive responses to stressors (excessive allostatic load)[10]. The hippocampus contains a large number of receptors for stress hormones such as glucocorticoids and is uniquely vulnerable to the negative effects of stressful experiences[6, 11]. Animal models have established that the hippocampal formation comprises multiple subfields with distinct histology, connectivity, and function[12]. Despite extensive examination in animals[13] that has found links between stress and the dentate gyrus subfield of the hippocampus, associations between hippocampal subfield integrity and psychological stress have not been studied in older humans[14]. Our focus on older adults is driven by the observation that hippocampal subfields are differentially affected by age across individuals. The causes and behavioral consequences of individual differences in hippocampal subfields among older adults are poorly understood. Our study sought to determine whether measures of psychological stress were related to these individual differences in hippocampal subfields among older adults. We hypothesize that higher levels of perceived stress will be associated with smaller total hippocampal volumes and hippocampal subfield volume (CA4/Dentate Gyrus (CA4/DG)) among a racially/ethnically diverse sample of nondemented community-dwelling older adults.

## Materials and Methods

### Participants

Between July 2011 and March 2014, a subset of 116 older adults was consecutively drawn from the Einstein Aging Study (EAS), an ongoing community-based volunteer sample of individuals over the age of 70 residing in Bronx, New York. EAS study design and methods have been described in detail elsewhere[15]. Briefly, potential participants were recruited through systematic sampling from Bronx County voter registration lists from the New York City Board of Elections. Participants were excluded who report severe sensory loss or medical conditions (including psychiatric symptomatology such as active hallucinations) that would interfere with completion of the in-house assessment, were non-English speakers, or were institutionalized. In addition to these exclusion criteria, participants were ineligible for the neuroimaging substudy if they were diagnosed with dementia or were ineligible for an MRI (e.g., unsafe metallic implants, claustrophobia). Dementia diagnosis was based on standardized clinical criteria from the Diagnostic and Statistical Manual, Fourth Edition (DSM-IV)[16] and required impairment in memory plus at least one additional cognitive domain as well as evidence of cognitive-related functional decline. Diagnoses were assigned at consensus case conferences attended by a study neurologist and neuropsychologist and included a comprehensive review of neuropsychological test results, relevant neurological signs and symptoms, and functional status. Written informed consent was obtained from all participants in accordance with study protocols approved by the Institutional Review Board of the Albert Einstein College of Medicine.

### Clinical Assessment

General cognitive status was assessed with the Blessed Information-Memory-Concentration test (BIMC; [17]). Scores range from 0 to 33, with higher scores reflecting greater global cognitive impairment. Depression was also assessed as previous studies have reported a relationship between depression and stress in older adults[18, 19] as well as depression and hippocampal integrity in older adults[20, 21]. Depressive symptoms were assessed using the 15-item Geriatric Depression Scale (GDS) in which participants respond either “yes” or “no” to 15 depressive symptoms experienced over the past week. Total GDS scores range from 0 to 15 with higher scores reflecting greater depressive symptomatology. Clinically significant depression was defined using a cut-off of 5 or greater [22].

### Stress Assessment

The 14-item Perceived Stress Scale (PSS-14) is a global assessment of an individual’s perception of psychological stress consisting of items that measure perceived stress experienced during the last month[23]. We selected the PSS-14 as a sensitive measure of chronic stress associated with ongoing life circumstances as well as possible future events. We have previously showed that the PSS-14 has a reliable factor structure and predictive validity in our larger older adult sample[18]. Additionally, we have shown that the PSS-14 measurement is stable over a 2-year period (ICC = 0.68) among older adults, demonstrating that it can capture the persistent nature of chronic stress.[5] The PSS-14 contains seven negative items (questions 1, 2, 3, 8, 11, 12, and 14) and seven positive items (questions 4, 5, 6, 7, 9, 10, and 13) with each item rated on a five point Likert-scale as follows: 0 = “never,” 1 = “almost never,” 2 = “sometimes,” 3 =“fairly often,” and 4 = “very often.” Total scores are calculated after reverse keying positive item scores and summing across items. Total scores range from 0 to 56, with higher scores reflecting greater perceived stress.

### MRI Acquisition

Imaging was performed using a 3.0 T MRI scanner (Achieva Quasar TX; Philips Medical Systems, Best, the Netherlands) and 32-channel head coil (Sense Head Coil; Philips Medical Systems). T1-weighted whole-head structural imaging was performed using sagittal three-dimensional magnetization-prepared rapid acquisition gradient echo (MPRAGE) with TR/TE 9.9/4.6ms; 240 mm^2^ field of view; 240×240 mm matrix; partition thickness, 1mm; and parallel acceleration factor 2.0.

### Image Processing

MRI data were processed with the FreeSurfer software package (http://surfer.nmr.mgh.harvard.edu/). Technical details of the development and validation of FreeSurfer have been previously described[24–26]. Briefly, skull stripping is achieved using a hybrid watershed/deformation procedure followed by automated Talairach transformation and segmentation of subcortical white and gray matter volumetric structures[26, 27]. FLAIR images were used for pial surface refinement. Hippocampal subfield volumes were measured based on methods described in Van Leemput and colleagues[28] that use Bayesian inference and a statistical model of the medial temporal lobe. The current analyses focused on four hippocampal subfields of interest: CA1, CA2/CA3, CA4/Dentate Gyrus (CA4/DG), and subiculum. The anatomical borders used to delineate these subdivisions are described fully in Van Leemput et al [28]. Segmentation results were visually inspected for errors in all datasets and manual edits were performed as needed. Entorhinal cortex (EC) and total hippocampal volume (HIP) were also obtained as primary MRI-derived variables of interest. The entorhinal cortex has been shown to be involved in the pathophysiology of Alzheimer’s disease[29, 30]. Whole brain (WB) volume was obtained as a control that was not thought to be associated with measures of perceived stress. Additionally, we controlled for differences in head size using estimated total intracranial volume (TICV) that was calculated based on procedures described in[31].

### Statistical Analyses

Statistical analyses were conducted using IBM SPSS Statistics for Windows, Version 22.0. (IBM Corp.; Armonk, NY). Age and education were examined as potential covariates using Pearson product-moment correlation coefficients. Race and gender were examined as potential covariates using Student’s t-tests. Pearson correlation coefficients were utilized to examine relationships between perceived stress and MRI-derived variables of interest (CA1, CA2/CA3, CA4/DG, subiculum, EC, HIP). Separate linear regression analyses were performed to examine perceived stress as an independent variable and MRI-derived variables of interest (CA1, CA2/CA3, CA4/DG, subiculum, EC, HIP) as dependent variables, with TICV, age, gender, and race as covariates. Linear regression was repeated with whole brain volume as a dependent variable to establish specificity of the findings.

## Results

### Sample Characteristics

Sample demographics, perceived stress, and MRI-derived volumes of interest are presented in **Table 1**. Due to unequal distributions of individuals across all ethnicity categories, we elected to focus on the dichotomous racial categories of “Caucasian” and “Not Caucasian.” As we did not have a priori laterality hypotheses, left and right volumes for all MRI variables were combined to reduce the number of statistical comparisons.

**Table 1:** Sample Demographics, Perceived Stress, and MRI Variables of Interest.

	Total Sample (N = 116)
Age, years, mean (SD)	79.42 (5.04)
Gender, %, women	63.8
Race, %, Caucasian	50.9
Education, years, mean (SD)	14.12 (3.38)
BIMC, total errors, median (range)	2.00 (0–13)
PSS-14, total score, mean (SD)	15.76 (7.85)
GDS, total score, mean (SD)	1.44 (1.52)
CA1 volume, mean (SD)	614.35 (70.42)
CA2/CA3 volume, mean (SD)	1696.07 (206.56)
CA4/Dentate gyrus volume, mean (SD)	956.77 (117.93)
Subiculum volume, mean (SD)	1060.85 (126.48)
Entorhinal cortex volume, mean (SD)	3220.69 (651.53)
Total Hippocampal volume, mean (SD)	6524.98 (806.00)
Whole brain volume, mean (SD)	981346.38 (103979.86)
TICV volume, mean (SD)	1343678.53 (204396.79)

*Note*: SD = Standard deviation; BIMC = Blessed Information-Memory-Concentration Test, score range 0–33; PSS-14 = Perceived Stress Scale– 14 item, score range 0–63; GDS = Geriatric Depression Scale, score range 0–15; TICV = Total Intracranial Volume. MRI volumetric data are given in cubic millimeters.

### Simple Correlations

Pearson correlation coefficients revealed that older age was associated with smaller brain volumes in HIP (r = -0.42, p≤0.01), CA2/CA3 (r = -0.32, p≤0.01), CA4/DG (r = -0.32, p≤0.01), and subiculum (r = -0.33, p≤0.01). Age was not associated with PSS-14 (r = 0.15, p = 0.12). Individuals with higher perceived stress had smaller regional brain volumes in HIP (r = -0.28, p≤0.01), CA2/CA3 (r = -0.26, p≤0.01), and CA4/DG (r = -0.26, p≤0.01) (**S1 and S3 Figs**). Men had larger volumes across all hippocampal regions of interest (range t = -2.92 to -5.06, p≤0.01all values) but gender was not associated with perceived stress (t = 1.43, p = 0.16). Education and race were not associated with the primary variables of interest, with the exception of Caucasians having larger CA1 subregion volumes (t = 2.57, p≤0.01). Because of their correlations with primary variables of interest, age, gender, and race were included as covariates in subsequent analyses.

### Perceived Stress and Hippocampal Subfield and Entorhinal Cortex Volumes

Linear regression models assessing the effect of perceived stress on hippocampal subfield and entorhinal cortex volumes are shown in **Table 2**. The model of the relationship of PSS-14 and CA2/CA3 volume controlling for TICV, age, gender, and race (F_(5, 115)_ = 9.33, p≤0.01, R^2^ = 0.30) revealed higher levels of perceived stress were associated with smaller CA2/CA3 volumes. The model of the relationship of PSS-14 and CA4/DG volume controlling for TICV, age, gender, and race (F_(5, 115)_ = 8.53, p≤0.01, R^2^ = 0.28) revealed higher levels of perceived stress were associated with smaller CA4/DG volumes. The contributions of additional covariates to these models are shown in **Table 2**. Models of associations between PSS-14 and CA1, subiculum, and EC volumes were not significant.

**Table 2:** Linear Regression Models Assessing the Effect of Perceived Stress on Hippocampal Subfield and Entorhinal Cortex Volumes.

	CA1			CA2/CA3			CA4/Dentate Gyrus			Subiculum			Entorhinal Cortex		
	Beta	t	p-value	Beta	t	p-value	Beta	t	p-value	Beta	t	p-value	Beta	t	p-value
PSS-14	-0.08	-1.05	0.30	-0.18	-2.24	**0.03**	-0.19	-2.28	**0.03**	-0.02	-0.19	0.85	0.04	0.45	0.65
Age	-0.12	-1.52	0.13	-0.27	-3.25	**≤0.01**	-0.26	-3.12	**≤0.01**	-0.31	-3.72	**≤0.01**	-0.07	-0.77	0.45
Gender	0.13	1.34	0.18	0.16	1.61	0.11	0.20	2.00	0.05	0.16	1.55	0.13	0.20	1.95	0.05
Race	-0.12	-1.58	0.12	-0.10	-1.25	0.22	-0.10	-1.15	0.26	-0.12	-1.36	0.18	-0.23	-2.74	**0.01**
TICV	0.45	4.77	**≤0.01**	0.24	2.41	**0.02**	0.18	1.75	0.08	0.25	2.44	**0.02**	0.27	2.64	**0.01**

*Note*: PSS-14 = Perceived Stress Scale; TICV = Total Intracranial Volume.

### Perceived Stress and Total Hippocampal and Whole Brain Volumes

Linear regression models assessing the effect of perceived stress on total hippocampal volume and whole brain volume are shown in **Table 3**. The model of the relationship of PSS-14 and total hippocampal volume controlling for TICV, age, gender, and race (F_(5, 115)_ = 8.35, p≤0.01, R^2^ = 0.28) revealed that higher levels of perceived stress were associated with smaller HIP and older age. The contributions of additional covariates to the model are shown in **Table 3**. The model of the association between PSS-14 and whole brain volume was not significant.

**Table 3:** Linear Regression Models Assessing the Effect of Perceived Stress on Total Hippocampal and Whole Brain Volumes.

	Hippocampus			Whole Brain		
	Beta	t	p-value	Beta	t	p-value
PSS-14	-0.20	-2.40	**0.02**	0.01	0.21	0.83
Age	-0.38	-4.53	**≤0.01**	-0.33	-7.36	**≤0.01**
Gender	0.10	1.03	0.31	-0.00	-0.06	0.95
Race	-0.07	-0.77	0.44	-0.00	-0.09	0.93
TICV	0.13	1.23	0.22	0.83	15.24	**≤0.01**

*Note*: PSS-14 = Perceived Stress Scale; TICV = Total Intracranial Volume.

### Perceived Stress, Hippocampal Regions of Interest, and Depressive Symptoms

Depressive symptoms were not normally distributed in our sample, with the majority of participants reporting one or no symptoms out of a total possible score of 15 (sample mean (SD) = 1.44(1.52)). Therefore, we elected to focus on the contribution of clinically significant depressive symptoms (GDS≥5) to our findings, with seven (6%) individuals meeting these criteria. All linear regression analyses were repeated with the removal of these individuals and the primary pattern of findings remained unchanged.

### Perceived Stress, Hippocampal Regions of Interest, and Global Cognitive Function

In order to assess the possible contribution of global cognitive function to our findings, Spearman’s correlations were performed to examine the relationship between BIMC and PSS-14 and the hippocampal regions of interest. Spearman’s correlations were chosen because the BIMC data were not normally distributed. BIMC was not correlated with PSS-14, but poorer performance on the BIMC was associated with smaller HIP (r_s_ = -0.18, p = 0.04), CA2/CA3 (r_s_ = -0.22, p = 0.02), CA4/DG (r_s_ = -0.19, p = 0.04), subiculum (r_s_ = -0.27, p≤0.01), and EC (r_s_ = -0.24, p = 0.01). All linear regression analyses were repeated with the inclusion of BIMC as a covariate and the primary pattern of findings remained unchanged.

## Discussion

Among older adults, higher levels of perceived chronic stress are associated with smaller volumes of CA4/dentate gyrus and CA2/CA3, subfields of the hippocampus that are thought to be uniquely sensitive to the modulation of stress[13]. We observed these relationships even after statistically controlling for age, gender, race, and after excluding individuals with significant depressive symptoms. Prior studies have linked HPA axis dysfunction with total hippocampal volume loss in clinical populations with Cushing’s syndrome[32], certain psychiatric disorders[33], including PTSD[34–36], and Alzheimer’s disease[37–39]. Lupien and colleagues reported that older adults with persistently elevated cortisol levels over a 5–6 year period had smaller hippocampal volumes and demonstrated poor performance on memory tasks, though stress was not directly measured[40]. Our study extends these findings by demonstrating a relationship between specific subfields of the hippocampus and psychological stress among older individuals living in the community.

Although prior human studies have measured total volume of the hippocampus, animal studies have demonstrated that the hippocampal formation is a heterogeneous structure with anatomically and functionally differentiated subfields[13] that may change differentially with age[41]. Input to the hippocampus occurs through the entorhinal cortex and then proceeds to the dentate gyrus, which in turn extends to the CA3 pyramidal neurons. The dentate gyrus is a region of sustained neurogenesis throughout adult life[41, 42]. Adult neurogenesis through neuronal cell proliferation, differentiation, and cell survival has been observed in most mammals, including humans[14, 43]. Neurogenesis in the dentate gyrus occurs in the subgranular zone where progenitor cells differentiate and eventually extend to CA3 pyramidal cells[13]. Neurogenesis declines with advanced age and may be associated with cognitive decline[14, 44].

Our finding that perceived stress is associated with preferential volume loss in CA4/DG and CA2/CA3 is largely consistent with animal models; both acute and chronic stress (e.g., electric shock, restraint, social isolation, predator odor) may inhibit cell proliferation, reduce neuronal differentiation and jeopardize new cell survival in the dentate gyrus[14, 42, 45]. Other findings indicate that perseveration of cells in the dentate gyrus is involved in HPA regulation and the development of anxiety-related behaviors, suggesting that neurogenesis in the dentate gyrus is integral to emotion regulation and to the pathophysiology of stress disorders[13], although some have cautioned against an oversimplification of this relationship [46]. Given the direct excitatory connections between dentate gyrus and CA3, it seems reasonable that dysfunction in the dentate gyrus may exert negative downstream effects on the CA2/CA3 subregion, a possibility supported by our observation of smaller volumes in this area. Proposed biological mechanisms for these relationships include elevated levels of circulating glucocorticoids released by stress-associated HPA activity and increased levels of cytokines such as interleukin-I[14, 47]. Our finding that higher perceived stress was associated with smaller CA4/DG and CA2/CA3 volumes but not entorhinal cortex is likely a reflection of our study sample that excluded persons with dementia. The entorhinal cortex is known to be involved in the pathophysiology of Alzheimer’s disease[29, 30]. Our observed pattern of stress and brain volume associations does not preclude a potential contribution of stress to the development of dementia, but rather suggests that stress may operate as an independent deleterious process in older adults even in the absence of other well-known neuropathological conditions.

As our study is cross-sectional, we are unable to determine causality; high levels of perceived stress may result in smaller hippocampal subfield volumes or, perhaps smaller hippocampal subfield volumes confer heightened vulnerability to the experience of stress in older adults. Future longitudinal studies are needed to fully examine this complex issue. In addition, in this study we used brain volumetric measures as a surrogate marker of neurodegeneration. However, measuring functional status of cell populations in these subregions requires further study using neuroimaging modalities such as fMRI, diffusion tensor imaging, or magnetic resonance spectroscopy imaging. Finally, our study focused on older adults. Future studies are needed to determine whether these same relationships would be observed in other age groups in individuals across the lifespan.

## Supporting Information

S1 Fig Perceived Stress and Total Hippocampal Volume among Older Adults. (JPG)

S2 Fig Perceived Stress and CA2/CA3 Volume among Older Adults. (JPG)

S3 Fig Perceived Stress and CA4/Dentate Gyrus Volume among Older Adults. (JPG)

## References

[1] Marin MF, Lord C, Andrews J, Juster RP, Sindi S, Arsenault-Lapierre G, et al Chronic stress, cognitive functioning and mental health. Neurobiology of learning and memory. 2011;96(4):583–95. 10.1016/j.nlm.2011.02.016 . 21376129

[2] Wilson RS, Barnes LL, Bennett DA, Li Y, Bienias JL, Mendes de Leon CF, et al Proneness to psychological distress and risk of Alzheimer disease in a biracial community. Neurology. 2005;64(2):380–2. 10.1212/01.WNL.0000149525.53525.E7 . 15668449

[3] Johansson L, Guo X, Waern M, Ostling S, Gustafson D, Bengtsson C, et al Midlife psychological stress and risk of dementia: a 35-year longitudinal population study. Brain. 2010;133(Pt 8):2217–24. 10.1093/brain/awq116 . 20488887

[4] Simard M, Hudon C, van Reekum R. Psychological distress and risk for dementia. Current psychiatry reports. 2009;11(1):41–7. . 19187707 10.1007/s11920-009-0007-z

[5] Munoz E, Sliwinski MJ, Scott SB, Hofer S. Global Perceived Stress Predicts Cognitive Change Among Older Adults. Psychology and aging. 2015 10.1037/pag0000036 . 26121285 PMC4559145

[6] McEwen BS. Sex, stress and the hippocampus: allostasis, allostatic load and the aging process. Neurobiol Aging. 2002;23(5):921–39. . 12392796 10.1016/s0197-4580(02)00027-1

[7] Lupien SJ, Evans A, Lord C, Miles J, Pruessner M, Pike B, et al Hippocampal volume is as variable in young as in older adults: implications for the notion of hippocampal atrophy in humans. Neuroimage. 2007;34(2):479–85. 10.1016/j.neuroimage.2006.09.041 . 17123834

[8] Pruessner JC, Baldwin MW, Dedovic K, Renwick R, Mahani NK, Lord C, et al Self-esteem, locus of control, hippocampal volume, and cortisol regulation in young and old adulthood. Neuroimage. 2005;28(4):815–26. 10.1016/j.neuroimage.2005.06.014 . 16023372

[9] Pruessner JC, Lord C, Meaney M, Lupien S. Effects of self-esteem on age-related changes in cognition and the regulation of the hypothalamic-pituitary-adrenal axis. Ann N Y Acad Sci. 2004;1032:186–90. 10.1196/annals.1314.017 . 15677407

[10] McEwen BS, Stellar E. Stress and the individual. Mechanisms leading to disease. Archives of internal medicine. 1993;153(18):2093–101. . 8379800

[11] Lupien SJ, McEwen BS, Gunnar MR, Heim C. Effects of stress throughout the lifespan on the brain, behaviour and cognition. Nature reviews Neuroscience. 2009;10(6):434–45. 10.1038/nrn2639 . 19401723

[12] Fanselow MS, Dong HW. Are the dorsal and ventral hippocampus functionally distinct structures? Neuron. 2010;65(1):7–19. 10.1016/j.neuron.2009.11.031 20152109 PMC2822727

[13] Fa M, Xia L, Anunu R, Kehat O, Kriebel M, Volkmer H, et al Stress modulation of hippocampal activity—spotlight on the dentate gyrus. Neurobiology of learning and memory. 2014;112:53–60. 10.1016/j.nlm.2014.04.008 . 24747273

[14] Schoenfeld TJ, Gould E. Stress, stress hormones, and adult neurogenesis. Experimental neurology. 2012;233(1):12–21. Epub 2011/02/02. 10.1016/j.expneurol.2011.01.008 21281629 PMC3715962

[15] Katz MJ, Lipton RB, Hall CB, Zimmerman ME, Sanders AE, Verghese J, et al Age-specific and sex-specific prevalence and incidence of mild cognitive impairment, dementia, and Alzheimer dementia in blacks and whites: a report from the Einstein Aging Study. Alzheimer disease and associated disorders. 2012;26(4):335–43. 10.1097/WAD.0b013e31823dbcfc 22156756 PMC3334445

[16] Diagnostic and Statistical Manual of Mental Disorders, DSM-IV. Washington, DC: American Psychiatric Association; 1994 p. 133.

[17] Blessed G, Tomlinson BE, Roth M. The association between quantitative measures of dementia and of senile change in the cerebral gray matter of elderly subjects. Brit J Psychiatry. 1968;114:797–811. 5662937 10.1192/bjp.114.512.797

[18] Ezzati A, Jiang J, Katz MJ, Sliwinski MJ, Zimmerman ME, Lipton RB. Validation of the Perceived Stress Scale in a community sample of older adults. Int J Geriatr Psychiatry. 2014;29(6):645–52. 10.1002/gps.4049 24302253 PMC4013212

[19] Payne C, Hedberg EC, Kozloski M, Dale W, McClintock MK. Using and interpreting mental health measures in the National Social Life, Health, and Aging Project. The journals of gerontology Series B, Psychological sciences and social sciences. 2014;69 Suppl 2:S99–116. 10.1093/geronb/gbu100 25360028 PMC4303090

[20] Ezzati A, Zimmerman ME, Katz MJ, Lipton RB. Hippocampal correlates of depression in healthy elderly adults. Hippocampus. 2013;23(12):1137–42. 10.1002/hipo.22185 23939871 PMC4018740

[21] Elbejjani M, Fuhrer R, Abrahamowicz M, Mazoyer B, Crivello F, Tzourio C, et al Depression, depressive symptoms, and rate of hippocampal atrophy in a longitudinal cohort of older men and women. Psychological medicine. 2015;45(9):1931–44. 10.1017/S0033291714003055 . 25896060

[22] Julian LJ, Gregorich SE, Earnest G, Eisner MD, Chen H, Blanc PD, et al Screening for depression in chronic obstructive pulmonary disease. COPD. 2009;6(6):452–8. Epub 2009/11/27. 10.3109/15412550903341463 19938969 PMC2981409

[23] Cohen S, Kamarck T, Mermelstein R. A global measure of perceived stress. Journal of health and social behavior. 1983;24(4):385–96. . 6668417

[24] Dale AM, Fischl B, Sereno MI. Cortical surface-based analysis. I. Segmentation and surface reconstruction. Neuroimage. 1999;9(2):179–94. 10.1006/nimg.1998.0395 . 9931268

[25] Fischl B, Salat DH, Busa E, Albert M, Dieterich M, Haselgrove C, et al Whole brain segmentation: automated labeling of neuroanatomical structures in the human brain. Neuron. 2002;33(3):341–55. . 11832223 10.1016/s0896-6273(02)00569-x

[26] Segonne F, Dale AM, Busa E, Glessner M, Salat D, Hahn HK, et al A hybrid approach to the skull stripping problem in MRI. Neuroimage. 2004;22(3):1060–75. 10.1016/j.neuroimage.2004.03.032 . 15219578

[27] Fischl B, Salat DH, van der Kouwe AJ, Makris N, Segonne F, Quinn BT, et al Sequence-independent segmentation of magnetic resonance images. Neuroimage. 2004;23 Suppl 1:S69–84. 10.1016/j.neuroimage.2004.07.016 . 15501102

[28] Van Leemput K, Bakkour A, Benner T, Wiggins G, Wald LL, Augustinack J, et al Automated segmentation of hippocampal subfields from ultra-high resolution in vivo MRI. Hippocampus. 2009;19(6):549–57. 10.1002/hipo.20615 19405131 PMC2739884

[29] Schroeter ML, Stein T, Maslowski N, Neumann J. Neural correlates of Alzheimer's disease and mild cognitive impairment: a systematic and quantitative meta-analysis involving 1351 patients. Neuroimage. 2009;47(4):1196–206. 10.1016/j.neuroimage.2009.05.037 19463961 PMC2730171

[30] Yang J, Pan P, Song W, Huang R, Li J, Chen K, et al Voxelwise meta-analysis of gray matter anomalies in Alzheimer's disease and mild cognitive impairment using anatomic likelihood estimation. Journal of the neurological sciences. 2012;316(1–2):21–9. 10.1016/j.jns.2012.02.010 . 22385679

[31] Buckner RL, Head D, Parker J, Fotenos AF, Marcus D, Morris JC, et al A unified approach for morphometric and functional data analysis in young, old, and demented adults using automated atlas-based head size normalization: reliability and validation against manual measurement of total intracranial volume. Neuroimage. 2004;23(2):724–38. 10.1016/j.neuroimage.2004.06.018 . 15488422

[32] Starkman MN, Gebarski SS, Berent S, Schteingart DE. Hippocampal formation volume, memory dysfunction, and cortisol levels in patients with Cushing's syndrome. Biol Psychiatry. 1992;32(9):756–65. . 1450290 10.1016/0006-3223(92)90079-f

[33] Bremner JD, Narayan M, Anderson ER, Staib LH, Miller HL, Charney DS. Hippocampal volume reduction in major depression. Am J Psychiatry. 2000;157(1):115–8. . 10618023 10.1176/ajp.157.1.115

[34] Gurvits TV, Shenton ME, Hokama H, Ohta H, Lasko NB, Gilbertson MW, et al Magnetic resonance imaging study of hippocampal volume in chronic, combat-related posttraumatic stress disorder. Biol Psychiatry. 1996;40(11):1091–9. 10.1016/S0006-3223(96)00229-6 8931911 PMC2910907

[35] Bremner JD, Randall P, Vermetten E, Staib L, Bronen RA, Mazure C, et al Magnetic resonance imaging-based measurement of hippocampal volume in posttraumatic stress disorder related to childhood physical and sexual abuse—a preliminary report. Biol Psychiatry. 1997;41(1):23–32. 8988792 10.1016/s0006-3223(96)00162-x PMC3229101

[36] Stein MB, Koverola C, Hanna C, Torchia MG, McClarty B. Hippocampal volume in women victimized by childhood sexual abuse. Psychological medicine. 1997;27(4):951–9. . 9234472 10.1017/s0033291797005242

[37] O'Brien JT, Ames D, Schweitzer I, Colman P, Desmond P, Tress B. Clinical and magnetic resonance imaging correlates of hypothalamic-pituitary-adrenal axis function in depression and Alzheimer's disease. Br J Psychiatry. 1996;168(6):679–87. . 8773809 10.1192/bjp.168.6.679

[38] De Leon MJ, George AE, Golomb J, Tarshish C, Convit A, Kluger A, et al Frequency of hippocampal formation atrophy in normal aging and Alzheimer's disease. Neurobiol Aging. 1997;18(1):1–11. . 8983027 10.1016/s0197-4580(96)00213-8

[39] de Leon MJ, McRae T, Tsai JR, George AE, Marcus DL, Freedman M, et al Abnormal cortisol response in Alzheimer's disease linked to hippocampal atrophy. Lancet. 1988;2(8607):391–2. . 2899791 10.1016/s0140-6736(88)92855-3

[40] Lupien SJ, de Leon M, de Santi S, Convit A, Tarshish C, Nair NP, et al Cortisol levels during human aging predict hippocampal atrophy and memory deficits. Nature neuroscience. 1998;1(1):69–73. 10.1038/271 . 10195112

[41] Small SA, Schobel SA, Buxton RB, Witter MP, Barnes CA. A pathophysiological framework of hippocampal dysfunction in ageing and disease. Nature reviews Neuroscience. 2011;12(10):585–601. Epub 2011/09/08. 10.1038/nrn3085 21897434 PMC3312472

[42] Epp JR, Chow C, Galea LA. Hippocampus-dependent learning influences hippocampal neurogenesis. Frontiers in neuroscience. 2013;7:57 Epub 2013/04/19. 10.3389/fnins.2013.00057 23596385 PMC3627134

[43] Cameron HA, Glover LR. Adult neurogenesis: beyond learning and memory. Annu Rev Psychol. 2015;66:53–81. 10.1146/annurev-psych-010814-015006 . 25251485 PMC5612417

[44] VanGuilder HD, Farley JA, Yan H, Van Kirk CA, Mitschelen M, Sonntag WE, et al Hippocampal dysregulation of synaptic plasticity-associated proteins with age-related cognitive decline. Neurobiology of disease. 2011;43(1):201–12. Epub 2011/03/29. 10.1016/j.nbd.2011.03.012 21440628 PMC3096728

[45] Hanson ND, Owens MJ, Nemeroff CB. Depression, antidepressants, and neurogenesis: a critical reappraisal. Neuropsychopharmacology. 2011;36(13):2589–602. Epub 2011/09/23. 10.1038/npp.2011.220 21937982 PMC3230505

[46] Petrik D, Lagace DC, Eisch AJ. The neurogenesis hypothesis of affective and anxiety disorders: are we mistaking the scaffolding for the building? Neuropharmacology. 2012;62(1):21–34. 10.1016/j.neuropharm.2011.09.003 21945290 PMC3698048

[47] Wang Z, Neylan TC, Mueller SG, Lenoci M, Truran D, Marmar CR, et al Magnetic resonance imaging of hippocampal subfields in posttraumatic stress disorder. Arch Gen Psychiatry. 2010;67(3):296–303. 10.1001/archgenpsychiatry.2009.205 20194830 PMC2848481

