# Supplementary figures and images for: Perceived Stress Is Differentially Related to Hippocampal Subfield Volumes among Older Adults
Source: PLoS One. 2016 May 4;11(5):e0154530. doi: 10.1371/journal.pone.0154530 (PMC4856349; doi:10.1371/journal.pone.0154530)

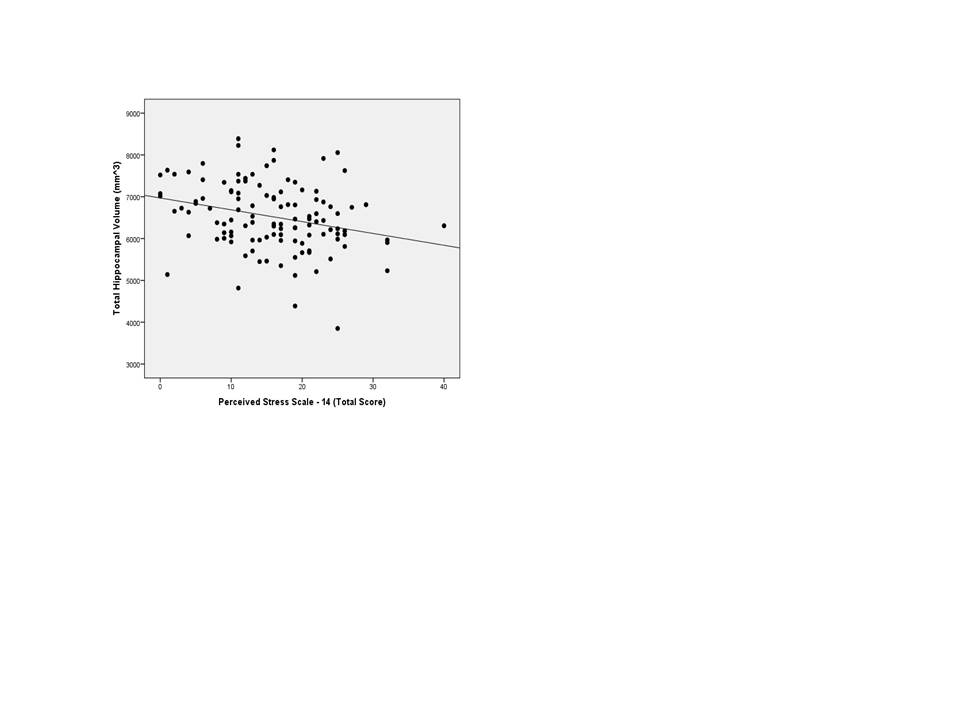

Supplement: S1 Fig — (JPG) [file pone.0154530.s001.jpg]

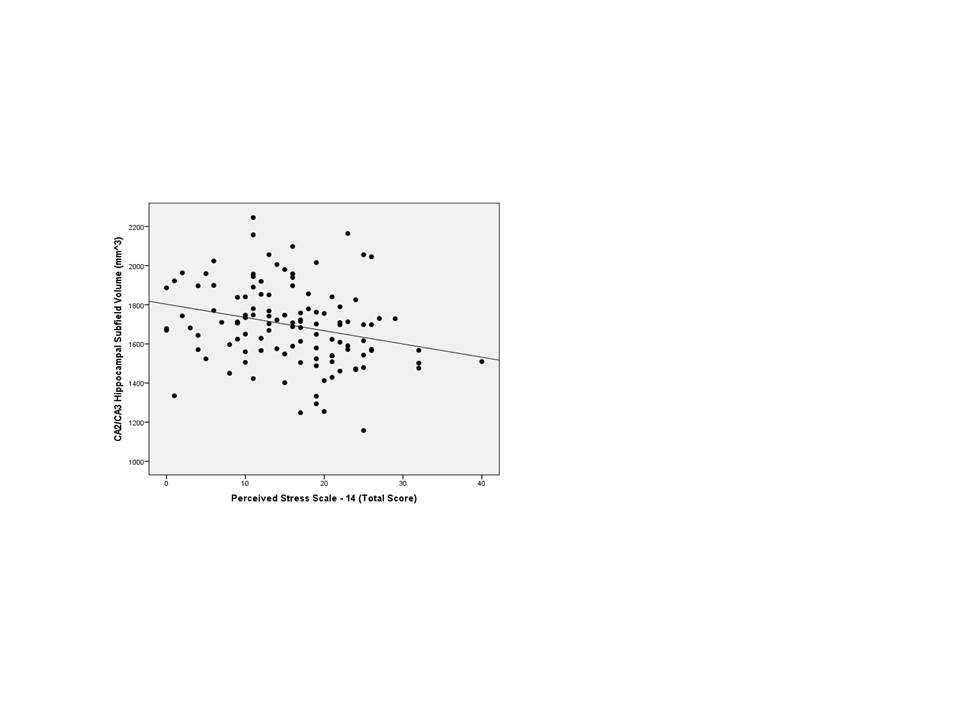

Supplement: S2 Fig — (JPG) [file pone.0154530.s002.jpg]

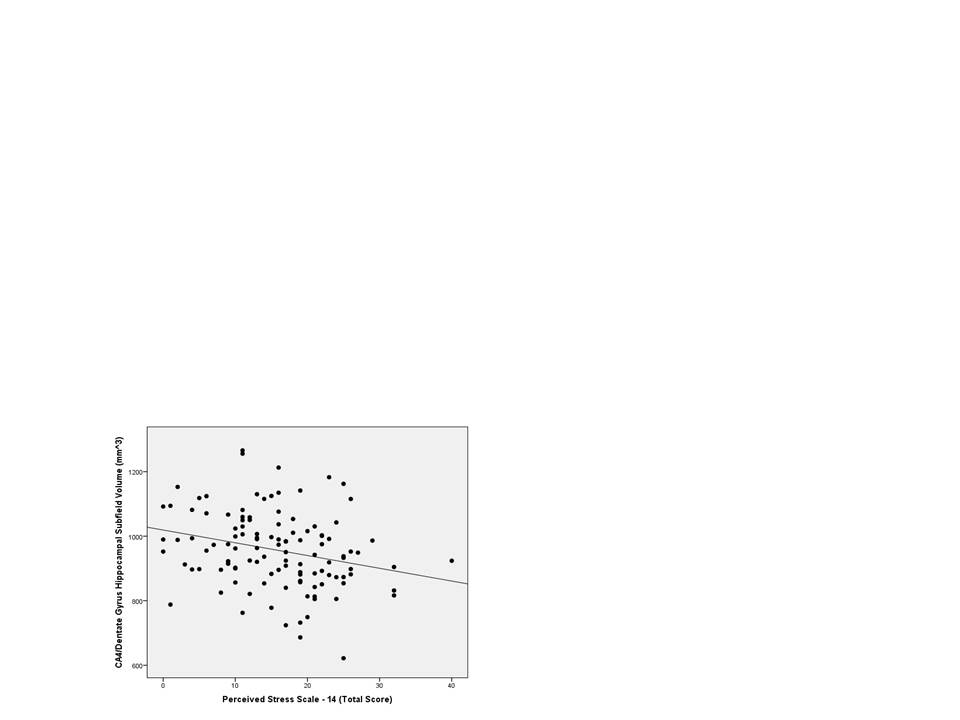

Supplement: S3 Fig — (JPG) [file pone.0154530.s003.jpg]
